# Job Demand, Job Control, and Impaired Mental Health in the Experience of Workplace Bullying Behavior: A Two-Wave Study

**DOI:** 10.3390/ijerph17041358

**Published:** 2020-02-20

**Authors:** Cristian Balducci, Elfi Baillien, Anja Van den Broeck, Stefano Toderi, Franco Fraccaroli

**Affiliations:** 1 Department of Psychology, University of Bologna, 40127 Bologna, Italy; stefano.toderi@unibo.it; 2 Department of Work and Organisation Studies, KU Leuven, 1000 Brussels, Belgium; elfi.baillien@kuleuven.be (E.B.); anja.vandenbroeck@kuleuven.be (A.V.d.B.); 3 Department of Psychosocial Science (TOPFORSK), University of Bergen, 5015 Bergen, Norway; 4 Optentia, North West University, Vanderbijlpark 1900, South Africa; 5 Department of Psychology and Cognitive Sciences, University of Trento, 38068 Rovereto, Italy; franco.fraccaroli@unitn.it

**Keywords:** workplace bullying, workplace harassment, emotional abuse in the workplace

## Abstract

Workplace bullying is an extreme social stressor at work leading to a severe deterioration of health amongst its targets. Research has revealed two important orders of factors that may trigger workplace bullying: Poor working conditions and individual factors such as impaired mental health that determine a personal psychological vulnerability to bullying. However, research has rarely investigated their role simultaneously. In response, we investigated whether the relationship between poor working conditions (i.e., high job demand) at time 1 (T1) and the experience of bullying at time 2 (T2) is strengthened by experiencing symptoms of impaired mental health at T1. We also tested whether job control—which contributes to better working conditions—at T1 moderates the relationship between job demand at T1 and bullying at T2. Participants (*N* = 235) were workers in the health sector. The time lag between T1 and T2 was one year. Cross-lagged path analysis revealed that the relationship between job demand at T1 and the experience of bullying behavior at T2 was strengthened by T1 impaired mental health. This suggests that considering both working conditions and individual factors together may be important for reaching a better understanding of the development of bullying.

## 1. Introduction

Workplace bullying is a peculiar form of interpersonal conflict in the workplace [1] defined as the systematic and persistent/repetitive experience of negative behavior at work, such as being withheld information that affects performance, being a target of rumors, or being socially isolated [2]. Workplace bullying has very negative consequences for the target’s mental health and for the work organization as well [3,4,5]. 

Research on the antecedents of bullying has revealed two main types of potential cause. First, bullying can be triggered by poor working conditions [6,7]. Research has shown, in fact, that work stressors such as role conflict, workload, and lack of social support are related to bullying, even prospectively [8,9,10]. Various processes have been proposed to explain the relationship between such stressors and the experience of bullying. For example, very poor working conditions may elicit stress reactions and feelings of frustration [11]. Distressed and frustrated employees may violate social norms through withdrawal or uncivil behavior (i.e., poor performance, rudeness), thus fueling retaliation by co-workers or superiors in the form of bullying. According to this view, then, bullying may be viewed as a strain-related phenomenon [12]. 

Second, research has shown that bullying may also be elicited by the target’s individual factors, and particularly by factors that act by instigating aggression by others [13]. For example, it has been argued that individuals high in negative affectivity and/or individuals experiencing anxiety and depressive and neurotic symptoms (i.e., individuals with impaired mental health) can “create or enact adverse circumstances through their own behavior,” p.190 [13]. They may, for example, perform less well in their jobs, be less able to manage their workflow, and contribute to the development of problems and conflicts at work, thus triggering aggression and bullying by others. There is also substantial evidence that other people may have a tendency to respond negatively to individuals with impaired mental health (e.g., [14]), strongly suggesting that impaired mental health may act as a psychological vulnerability factor for becoming a target of bullying. In line with this view, as observed by Nielsen and Einarsen [15], a recurrent finding in longitudinal studies on the cause–effect associations between workplace bullying and mental health problems is that existing mental health symptoms and problems are not only a consequence of bullying; they also predict the later experience of bullying (e.g., [16]).

Strikingly, studies on the antecedents of bullying focusing on poor working conditions and on individual factors of targets have developed largely independently of each other. However, it is unlikely that work–environmental and individual factors do not interact in predicting the experience of bullying. This idea corresponds to suggestions by scholars in the field of workplace bullying, who have underlined its multicausal nature and called for studies that combine both working conditions and individual factors [15]. The current study aims to test this view.

Specifically, to further investigate the role of poor working conditions in the experience of bullying, in the present study, we made use of the widely known job demand-control (JDC) model [17]. The central idea of this model is that the psychological job demand—which mainly refers to the amount of work to be done, pace of work, and time pressure—induces mental alertness and arousal and the experience of work-related strain. Therefore, by fueling work-related strain and the associated processes (e.g., poor performance—see above), it is highly likely that the psychological job demand is related to the experience of bullying. By contrast, job control, which concerns authority over decisions about one’s work and skills utilization, is believed to act as a resource that offsets the job demand–strain relationship. Thus, job control is likely to protect from the experience of bullying. 

We therefore hypothesized that:

**Hypothesis 1**: *Job demand would be positively related to the subsequent experience of bullying.*

**Hypothesis 2**: *The relationship between job demand and the subsequent experience of bullying would be moderated by job control, so that when job control is higher, the relationship between job demand and bullying would be weaker.*

Moreover, by combining the evidence on the work environment and individual factors as antecedents of bullying, we expected to find that impaired mental health would act not only as a cause for subsequent bullying per se but also as a strengthening factor in the job demand/bullying relationship. There is a paucity of studies on the moderating role of impaired mental health in the path leading to bullying (see [18], for an exception). Nevertheless, we hypothesized that when confronted with poor working conditions, workers, in particular, experiencing symptoms of impaired mental health may report bullying. Generally, individuals with impaired mental health perceive themselves as less self-efficacious and as having less control over situations [19,20]. Thus, the tension and arousal normally generated by a high job demand may be amplified in such individuals, leading them to more easily enact the additional distress experienced through improper behavior (e.g., constantly complaining, poor performance), thus giving reasons to others to aggress or marginalize them. We accordingly formulated the following additional hypotheses:

**Hypothesis 3**: *Impaired mental health would be positively related to subsequent bullying.*

**Hypothesis 4**: *The relationship between job demand and the subsequent experience of bullying would be moderated by impaired mental health. Specifically, at higher levels of impaired mental health, the relationship between job demand and subsequent bullying would be stronger.*

To test the proposed hypotheses, we implemented a two-wave full panel design. We focused on a sample of workers of the healthcare sector, which is a high-risk sector as far as bullying is concerned [21]. Moreover, research has demonstrated that the healthcare sector is characterized by highly stressful working conditions such as time pressure [22], long working hours [23], and motional demands [24]. Such distressing working conditions may be an advantage to study a strain-related phenomenon such as workplace bullying.

## 2. Materials and Methods

### 2.1. Procedure and Participants

We conducted a two-wave study among health-sector employees working for a National Health Service (NHS) agency in Italy. We selected a one-year time lag in line with findings in occupational health research that have indicated a one-year lag as appropriate for demonstrating causal relationships between stressors and strain [25]. The data were collected using a paper-and-pencil self-report questionnaire administered during working hours on a voluntary basis. No ethical approval was sought as the study was part of a mandatory risk assessment project conducted by the focused organization under the Italian health and safety law (Legislative Decree n. 81/2008). However, the study was conducted in line with the Helsinki Declaration [26], as well as the Italian data protection regulation (Legislative Decree n. 196/2003). The data from the two waves were matched by means of an anonymous personal code created by the participants using factual personal information. On request by the organization, only employees of the departments most at risk of work-related stress according to a number of indicators (e.g., sickness absence, turnover, disciplinary actions) established by the Italian health and safety law were invited at both times. At T1, a total of 574 employees returned the questionnaire (response rate = 75.4%). The T2 questionnaire was completed by 508 employees (response rate = 65%). Matching of the data from the two surveys was possible for 235 participants (response rate relative to T1 = 40.9%). The relatively low response rate obtained is not unusual in this area of research [27]. 

The final sample included 86.3% females, which corresponds to the healthcare sector having predominantly female employees (http://www.who.int/hrh/statistics/spotlight_2.pdf). The majority of the respondents were between 30 and 39 years of age (37.3%) or between 40 and 49 years of age (37.8%). Regarding occupation, most of participants were nurses (72.7%) or administrative staff (18.2%). Medical doctors and other workers (e.g., cleaning personnel) were also represented. About 83% of the participants had a job tenure of more than 5 years. Almost all participants (97%) had a permanent job contract. With the exception of an underrepresentation of medical doctors, the main characteristics of the final sample (gender, age, tenure, and job contract) correspond sufficiently well with those of the workforce of the surveyed NHS Agency. 

### 2.2. Measures

We measured the experience of bullying behavior by using a shortened version of the Negative Acts Questionnaire-Revised (NAQ-R; [28]), which has been specifically validated in Italy [29]. This version includes nine items investigating how often the respondent has experienced a variety of negative behaviors at work during the last six months (e.g., “Your work and effort have been persistently criticized”). Responses to items were made on a 5-point scale ranging from 1 (“Never”) to 5 (“Daily”).

Job demand was measured by using five items (e.g., “I have to work very fast”) from the Job Content Questionnaire (JCQ; [17]). Responses were given on a 4-point scale ranging from 1 (“Strongly disagree”) to 4 (“Strongly agree”). 

Job control was measured using eight items (e.g., “In the organization of my work I have a lot to say”) of the JCQ decision latitude dimension. Again, responses ranged from 1 (“Strongly disagree”) to 4 (“Strongly agree”). 

Building on some previous studies in this area [9], we measured impaired mental health by using the 12-item version of the General Health Questionnaire (GHQ-12; [30]). The GHQ-12 investigates the respondent’s experience of a number of psychological symptoms such as anxiety and depressive symptoms (e.g., “You have been capable of making decisions”), with responses ranging from 0 (“No” or “More than usual,” according to specific items) to 3 (“Much more than usual” or “Much less than usual”). We adopted the widely known conventional scoring method for the GHQ items (i.e., 0-0-1-1) whereby each item is scored in terms of presence vs. absence of the investigated symptom. Thus, the GHQ total score varied from 0 to 12.

### 2.3. Data Analysis

The hypotheses were tested by conducting cross-lagged path analysis with the software Mplus 7.4 (Muthen and Muthen, Los Angeles, CA, U.S.A). All the information available from the 235 participants was used to fit the different tested models (i.e., no listwise deletion of cases was implemented (On this, please see: http://www.statmodel.com/discussion/messages/11/11024.html?1469626359)). For each hypothesis, we first developed an appropriate baseline model and then freed the path (or paths) associated with the hypothesis (or hypotheses) tested, so that we assessed both the significance of the freed path (or paths) and whether the resulting model provided a significantly better fit to the data than the baseline model. More details on the different models tested are provided below. 

Model fit was assessed according to the following criteria: χ^2^ statistic, comparative fit index (CFI), Tucker−Lewis index (TLI), the root-mean-square error of approximation (RMSEA), and the standardized root-mean-square residual (SRMR). The critical value of χ^2^ is sensitive to sample sizes [31]. As standard practice, we accepted TLI and CFI values greater than 0.90 and RMSEA and SRMR values lower than 0.08 [see 31]. Preliminary analyses were conducted by using SPSS 22 (IBM, Armonk, NY, United States).

## 3. Results

As participants in the sample were clustered in 16 different departments, before conducting the main analysis, we calculated the amount of variance in T2 bullying explained by departmental membership. The results of this preliminary analysis revealed that 1.5% of variance (i.e., Intraclass Correlation Coefficient = 0.015) was accounted for by departmental membership, making it practically inconsequential to control for the hierarchical structure of the data [32]. 

Additionally, we carried out an attrition analysis to test whether drop-out at T2 was related to the main study variables, thus causing a selection bias [33]. To this end, we conducted a logistic regression analysis on the 574 participants in the T1 survey, in which we predicted drop-out from the study (0 = participation in follow-up; 1 = drop out). The predictors were gender (0 = male; 1 = female), age (0 = less than 40 years; 1 = 40 years or more), occupational position (0 = all positions except nurse; 1 = nurse), tenure (0 = five years or less; 1 = more than five years), and the main study variables (the experience of bullying behavior, job demand, job control, and impaired mental health). Our results revealed that nurses (Odds Ratio (OR) = 0.53, *p* < 0.01) and female participants (OR = 0.46, *p* < 0.01) were less likely to drop out, while reporting a greater job demand positively related to drop-out (OR = 1.70, *p* < 0.05). None of the other variables were related to drop-out. These results show that drop-out is unlikely to have biased our results, as selection bias only occurs if drop-out from the study is related to both the predictor(s) and the outcome variable [33].

Means, standard deviations, correlations, and Cronbach’s alphas of the study variables are reported in Table 1. The mean level of bullying behavior at both T1 and T2 was low, falling between the response category “Never” and “Rarely” on the 1–5 response scale adopted. However, this is the level usually found in organizational studies of bullying [7]. To be noted is the high stability of the bullying variable between the baseline survey and follow-up (r = 0.66). In addition, T1 and T2 job demand and T1 and T2 impaired mental health showed significant correlations with T2 bullying, while the correlation between the same variables and T1 bullying were weaker. Job control, the socio-demographic variables (i.e., age and gender), tenure, and organizational position did not show significant correlations with bullying.

**Figure 1:**
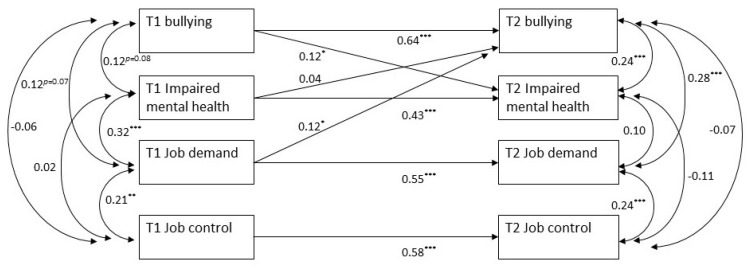
Path analytic model testing the impact of T1 job demand and T1 impaired mental health on T2 bullying. Note. * *p* < 0.05. ** *p* < 0.01. *** *p* < 0.001.

We then ran cross-lagged path analysis to test the study hypotheses. The path analytic model first fit to the data (M1) acted as the baseline model for testing Hypothesis 1 and Hypothesis 3 that, respectively, T1 job demand and T1 impaired mental health would impact T2 bullying. In this model, all the main study variables at T2 (i.e., bullying, job demand, job control, and impaired mental health) were predicted by their respective T1 level. Additionally, this model also included the correlations between the four variables at both T1 and T2, as well as a path from T1 bullying to T2 impaired mental health. The latter path accounted for a well-established influence of bullying on symptoms of impaired mental health. This model fit the data well (χ^2^ (11) = 16.77, *p* = 0.11; CFI = 0.99; TLI = 0.97; RMSEA = 0.047 [CI = 0.001–0.090]; SRMR = 0.054), with the path from T1 bullying to T2 impaired mental health being positive and significant (std. path = 0.13, *t* = 2.024, *p* < 0.05). We then fit a second model (M2) in which, in addition to the paths of the baseline model, the paths from T1 job demand to T2 bullying and from T1 impaired mental health to T2 bullying were also included. The resulting model fit the data well (χ^2^ (9) = 9.51, *p* = 0.39; CFI = 0.999; TLI = 0.997; RMSEA = 0.016 [CI = 0.001–0.076]; SRMR = 0.034) and significantly better than the baseline model (∆χ^2^ (2) = 7.26, *p* < 0.05). In this model (i.e., M2), which is graphically represented in Figure 1, the path from T1 job demand to T2 bullying was positive and significant (std. path = 0.12, *t* = 2.218, *p* < 0.05), while the path from T1 impaired mental health to T2 bullying was not significant (std. path = 0.04, *t* = 0.787, ns). These results supported Hypothesis 1, while they did not support Hypothesis 3.

To check for the potential effect from T1 bullying to T2 job demand (i.e., reverse causation), we tested a further model (M3). M3 included the T1 bullying-T2 job demand path in addition to the paths included in M1. Results showed that M3 did not fit better than M1 (∆χ^2^ (1) = 2.127, ns) and that the path from T1 bullying to T2 job demand was not significant (std. path = 0.08, *t* = 1.567, ns), thus excluding reverse causation. 

To test for the moderation hypothesis that T1 job control would attenuate the T1 job demand-T2 bullying relationship (Hypothesis 2), we proceeded by first building an appropriate baseline model. This model (M4) included, in addition to the variables and paths included in M1 (see above), the T1 job demand by the T1 job control interaction variable, which was allowed to correlate with the other T1 variables, and the paths T1 job demand-T2 bullying and T1 job control-T2 bullying. This model fit the data well, χ^2^ (13) = 14.873, *p* = 0.32; CFI = 0.995; TLI = 0.989; RMSEA = 0.025 [CI = 0.001–0.072]; SRMR = 0.035. The path T1 job demand-T2 bullying was positive and significant (std. path = 0.14, *t* = 2.692, *p* < 0.01), while the path T1 job control-T2 bullying was not significant (std. path = −0.04, *t* = 0.666, ns). We then fit an additional model (M5) with the same variables and paths as M4 and an additional path from the interaction variable (T1 job demand by T1 job control) to T2 bullying. M5 fit the data well, χ^2^ (12) = 12.063, *p* = 0.44; CFI = 0.999; TLI = 0.999; RMSEA = 0.005 [CI = 0.001–0.067]; SRMR = 0.034, but not significantly better than M4 (∆χ^2^ (1) = 2.81, *p* > 0.05). The interaction path T1 job demand by T1 job control was negative (as expected) but did not reach the significance level (std. path = −0.09, *t* = −1.693, *p* = 0.09). Thus, we did not find evidence in line with Hypothesis 2.

**Figure 2:**
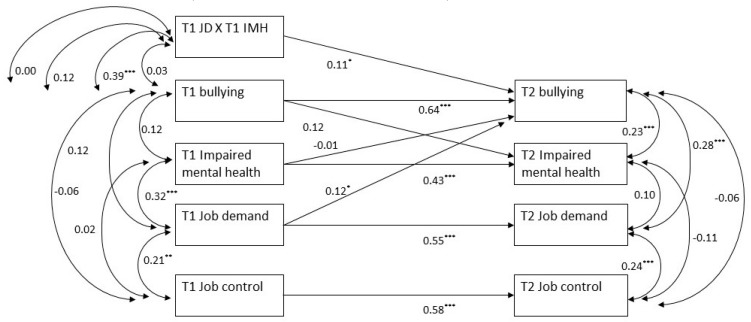
Path analytic model testing the interaction between T1 job demand and T1 impaired mental health (T1 JD X T1 IMH) on T2 bullying. Note. *p < 0.05. ** *p* < 0.01. *** *p* < 0.001.

**Figure 3:**
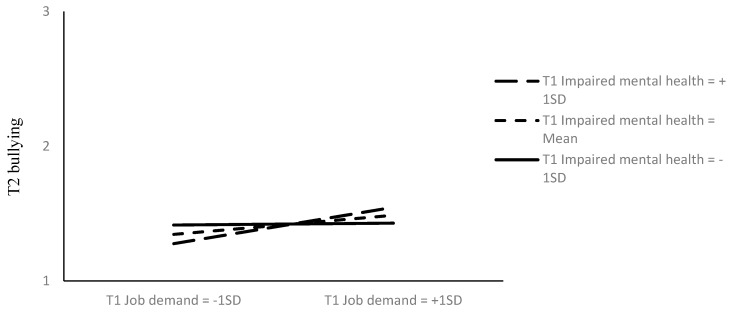
Interaction between T1 job demand and T1 impaired mental health in the prediction of T2 bullying. Note. SD = standard deviation.

Finally, to test for Hypothesis 4 that the T1 job demand-T2 bullying relationship would be strengthened by T1 impaired mental health, we followed the same procedure already used for testing Hypothesis 2. In this case, the baseline model (M6) was characterized by the paths and variables of M1 above, plus the T1 job demand by the T1 impaired mental health interaction variable, which was allowed to correlate with the other T1 variables. M6 also included the paths T1 job demand-T2 bullying and T1 impaired mental health-T2 bullying. M6 fit the data well, χ^2^ (13) = 16.384, *p* = 0.23; CFI = 0.992; TLI = 0.981; RMSEA = 0.033 [CI = 0.001−0.077]; SRMR = 0.036. The path T1 job demand-T2 bullying was positive and significant (std. path = 0.12, *t* = 2.216, *p* < 0.05), while the path T1 impaired mental health-T2 bullying was not significant (std. path = 0.04, *t* = 0.789, ns). Subsequently, we freed the interaction path T1 job demand by T1 impaired mental health to T2 bullying and found that the resulting model (M7) fit the data well (χ^2^ (12) = 11.689, *p* = 0.47; CFI = 0.999; TLI = 0.999; RMSEA = 0.001 [CI = 0.001−0.065]; SRMR = 0.033) and significantly better than M6 (∆χ^2^ (2) = 4.695, p < 0.05). The interaction path was positive and significant (std. path = 0.11, *t* = 2.175, *p* < 0.05) and explained 1.2% additional variance in T2 bullying. A graphical representation of M7 is shown in Figure 2 and the plot of the interaction T1 job demand by T1 impaired mental health on T2 bullying is given in Figure 3. From Figure 3, it can be seen that there was a stronger relationship between T1 job demand and T2 bullying at higher levels of impaired mental health (1 SD above the mean, b = 0,135, p < 0.01), as compared to when T1 impaired mental health was lower (1 SD below the mean, b = 0.007, ns).

As a last step, we repeated the main analyses by including age, gender, tenure, and position (see Table 1) as uncorrelated covariates affecting T2 bullying (i.e., the main study-dependent variable) and found that the results were not substantially affected. These additional analyses are available upon request from the first author.

## 4. Discussion

Most research on the antecedents of workplace bullying has focused on either working conditions [34,35] or individual factors [27]. As a consequence, little is known about person/environment interactions as triggers of bullying. The main aim of our research was to focus on this interaction by using a two-wave full panel design with a one-year time lag. The results support the idea that the experience of bullying behavior may be a strain phenomenon, being triggered by poor working conditions that have been extensively associated with work-related stress, especially in the case of employees with impaired mental health. As such, our study has shed further light on the multi-causal nature of workplace bullying and on the synergistic effects between its different causes. 

Specifically, in line with our first hypothesis, we found that job demand was positively related to the experience of bullying as reported one year later. The percentage of variance uniquely explained by job demand on subsequent bullying was not very high (i.e., 2.6% when controlling for job control and 2.0% when controlling for psychological vulnerability, as indicated by additional analyses). However, it was higher than that reported in previous research by Baillien et al. [8], who found that job demand and job control together explained 1% additional variance in bullying. Such a low percentage of variance, however, should not be underestimated given the multi-causal nature of bullying. According to Cohen et al. ([36], p. 151), even 1–2% of explained variance in the criterion may represent a material effect, despite the customary disparagement of effects of this magnitude. 

Overall, our finding complies with the prevailing idea in the workplace bullying research domain that working conditions may have an impact on being the target of bullying through different processes, such as by inducing the distressed employee to behave improperly at work, which may cause others to react aggressively toward him/her [15].

Our research did not provide evidence for a moderating role of job control on the job demand−bullying relationship. Following the JDC model [17] and previous research on bullying [8], we reasoned that autonomy and the possibility to use one’s own skills and competences at work would buffer the development of bullying from job demand. This is because job control may act as a resource, thus reducing the strain experiences generated by a high job demand, which may be critical in activating the processes leading to bullying. However, it is to be noted that, while the anti-strain role of job control is a central tenet of the demand-control model, it has not been consistently supported empirically [37]. It may also be that, in the case of workplace bullying, other kinds of resources play a more important role. For example, the position taken by the colleagues of the targets has been considered critical in the escalation of bullying [38]. Thus, social support may be more important than job control as a buffering resource. 

Attesting to the interplay between poor working conditions and individual factors in predicting bullying, our research showed that impaired mental health strengthened the role of job demand on the experience of bullying. Previous research on personal factors in bullying has either been descriptive in nature [39] or has investigated the potential main effect of such factors (e.g., [40]. Furthermore, almost all research has been cross-sectional in nature, with the consequence that the true causal direction of the postulated relationship is unclear. 

Our results suggest that high job demand is a cause of bullying particularly for employees who report more symptoms of impaired mental health. The results are in line with the idea that individuals with impaired mental health have fewer personal resources (e.g., energy, self-control, assertiveness, and other coping skills) to deal with the tension generated by a high job demand. This, in turn, may lead them to experience more difficulties in regulating their behavior and emotional expression in interpersonal relationships. Thus, individuals with impaired mental health may more easily experience the behavioral and psychological processes (e.g., violation of social norms, underachievement) that lead to an employee becoming a target of bullying. 

The findings of the present study should be considered in light of a number of methodological issues and limitations. The first issue concerns the relatively small sample of employees available for the lagged analyses. The final sample size was determined mainly by attrition and by the fact that some participants did not report the personal code we used to match the data of the two surveys. The personal code consisted of easy-to-remember factual personal information (e.g., the first two letters of the surname of the participant’s mother) and that, in principle, did not violate anonymity. We also emphasized anonymity and confidentiality on the first page of the adopted questionnaire. Nevertheless, given the sensitivity of the issues investigated in the survey (i.e., bullying, mental health symptoms), it may be that some participants did not perceive that the necessary safety of such personal information was in place. It should be noted, however, that samples like the one that we had available are not uncommon in this area of research [27]. Furthermore, despite the sample size, we were able to demonstrate a synergistic effect between different potential causes of bullying (i.e., work environmental and individual factors), which is a strength of the study as such kinds of effects have rarely been demonstrated (see [15]).

The second issue regards the target’s perspective that we have adopted in studying bullying, which is the current practice in this area of research. An alternative would be to adopt the actor’s perspective, which may have some advantages. For example, in the investigation of the causes of bullying, the actor’s perspective may help in identifying working conditions that lead to the enactment of bullying [41]. 

Another issue relates to the time lag adopted in our study. On considering previous research, we did not find consistency with regard to the adopted time lag. Time lags of six months [8], one year [9], and two years [42] have been adopted. With shorter time lags, evidence was found for an effect of working conditions on bullying, whilst with longer time lags, such evidence did not emerge, and reversed causation was indeed supported [42]. Thus, from the still few multi-wave studies, including the present one, it seems that the optimal time lag to detect the effect of working conditions on bullying is between six months and one year. 

A further limitation has to do with the generalizability of the emerged findings, as the sample was made by employees of the healthcare sector, who tend to experience high levels of bullying and distressing working conditions. Employees of the healthcare sector are also prevalently females, and the female gender is a risk factor for being the target of bullying [7]. Thus, at most, the emerged findings may be generalizable to healthcare sector work contexts. 

As a final limitation, all the variables of our study were self-reported, so their relationships may have been inflated by the common method bias [43]. Multisource studies are needed, although it is very difficult to implement such studies due to the very sensitive nature of the bullying topic. 

## 5. Conclusions

Despite the limitations described above, we believe our study contributes to the growing body of evidence on the role of working conditions and individual factors as antecedents of bullying. It makes an original contribution to the field by exploring possible interaction effects between the person and his/her work environment, which is an underinvestigated topic. The results highlight the possibility of using different preventive strategies to forestall bullying escalation. First and most importantly, our study suggests that interventions on working conditions (e.g., monitoring and modulating the job demand) may prevent the escalation of workplace bullying. Our results also suggest that employees with impaired mental health may be the targets of preventive interventions (e.g., on self-care or assertiveness), especially in poor working conditions—when they can more easily become targets of bullying. Recent work suggests that managers play an important role in preventing work-related strain [44]. Managers could, for example, be trained first to recognize workers experiencing symptoms of impaired mental health and then to monitor and manage the manifestations of their strain reactions. This should help decrease the risk of these workers becoming targets of aggression and bullying. 

## Figures and Tables

**Table 1:** Properties and Pearson’s correlations of study variables—Cronbach’s alpha on the diagonal, where appropriate (N varying from 197 to 233).

Variable	*M*/%	*SD*	1	2	3	4	5	6	7	8	9	10	11
1- T2 Bullying	1.45	0.58	(0.89)										
2- T1 Bullying	1.42	0.49	0.66 ***	(0.85)									
3- T2 Impaired mental health	2.00	2.70	0.33 ***	0.17 *	(0.86)								
4- T1 Impaired mental health	1.79	2.45	0.18 **	0.12	0.45 ***	(0.83)							
5- T2 Job demand	2.95	0.44	0.36 ***	0.14 *	0.26 ***	0.21 **	(0.70)						
6- T1 Job demand	2.88	0.43	0.21**	0.12	0.30 ***	0.32 ***	0.56 ***	(0.65)					
7- T2 Job control	3.08	0.33	−0.06	−0.02	−0.02	0.02	0.23 **	0.11	(0.70)				
8- T1 Job control	3.05	0.36	−0.07	−0.07	0.04	0.01	0.13	0.22 **	0.59 ***	(0.74)			
9- Gender ^a^ (% female)	86.3	-	−0.11	−0.08	0.15*	0.10	−0.10	0.02	0.04	0.02			
10- Age ^b^ (% ≥ 40 years)	51.1	-	0.06	0.04	−0.08	0.02	−0.15 **	−0.13	−0.16 *	−0.20 **	−0.02		
11- Tenure ^c^ (% > 5 years)	83.0	-	0.07	−0.04	0.06	0.06	0.07	0.02	−0.04	−0.10	−0.12	0.33 ***	
12- Position ^d^ (% nurse)	72.7	-	−0.01	−0.10	0.07	0.03	0.26 ***	0.14 *	0.30 ***	0.31 ***	0.09	−0.20 **	0.09

Note. ^a^ 0 = male; 1 = females; ^b^ 0 = less than 40 years; 1 = 40 years or more; ^c^ 0 = 5 years or less, 1 = more than 5 years; ^d^ 0 = All positions except nurse, 1 = Nurse; * *p* < 0.05. ** *p* < 0.01. *** *p* < 0.001.
